# A new fractional Cattaneo model for enhancing the thermal performance of photovoltaic panels using heat spreader: energy, exergy, economic and enviroeconomic (4E) analysis

**DOI:** 10.1007/s11356-023-29654-8

**Published:** 2023-09-18

**Authors:** Eman F. El-Gazar, Hamdy Hassan, Sherif I. Rabia, Changhong Hu, Waheed K. Zahra

**Affiliations:** 1 Basic Science Department, Benha Faculty of Engineering, Benha University, Benha, Egypt; 2 Department of Mathematics, Institute of Basic and Applied Sciences, Egypt-Japan University of Science and Technology (E-JUST), New Borg El-Arab City, Alexandria, Egypt; 3 Mechanical Engineering Department, Faculty of Engineering, Assiut University, Assiut, Egypt; 4 Energy Resources Engineering Department, Egypt-Japan University of Science and Technology (E-JUST), Alexandria, Egypt; 5 Department of Engineering Mathematics and Physics, Faculty of Engineering, Alexandria University, Alexandria, Egypt; 6 Research Institute for Applied Mechanics, Kyushu University, Fukuoka, Japan; 7 Department of Engineering Physics and Mathematics, Faculty of Engineering, Tanta University, Tanta, Egypt

**Keywords:** Cattaneo model, Riemann–Liouville fractional derivative, PV system, Heat spreader, Exergoeconomic, Enviroeconomic

## Abstract

A new fractional non-Fourier (Cattaneo) photovoltaic (PV) model is presented to enhance the thermal performance of a PV system combined with a heat spreader (HS). The fractional Cattaneo model is shown to be effective in examining transient processes across the entirety of a PV system, in contrast to the conventional Fourier model’s inability to predict system performance. Consequently, a comparison is conducted between the classical Fourier model with the fractional Fourier and fractional Cattaneo models for the PV system. The impact of using an aluminum heat spreader, with rectangular and trapezoidal shapes, has been developed under hot and cold climate conditions. The findings show that adding a trapezoidal heat spreader reduced the cell temperature by 20 K in summer and 12 K in winter. The reduction in the PV temperature led to an enhancement in daily average power by approximately 28% and 37% in hot and cold weather, respectively. Moreover, economic, exergoeconomic, and enviroeconomic assessment is introduced. The outcomes revealed that the electrical production costs of the rectangular and trapezoidal HS systems are 0.272 and 0.214 $/kWh, respectively, while about 0.286 $/kWh for the conventional PV panel. Based on the environmental study, the estimated CO_2_ reduction for PV, PV with rectangular HS, and PV with trapezoidal spreader is 0.5504, 0.7704, and 0.8012 tons, respectively. Finally, real experimental data are used to validate the fractional Cattaneo model. The results demonstrate that there is a great fitting with the measured data, with errors in PV power and exergy efficiency of just 0.628% and 3.84%, respectively, whereas their corresponding values for the classical model are 5.72 and 13.13%.

## Introduction 

Nowadays, the usage of sustainable energy is becoming more popular, due to the growth in the human population and the attention to environmental issues. As a result, solar energy is considered one of the most widely spread renewable energy sources (Ahmadi et al. 2017). Today, electricity from solar cells has become cost-competitive in many countries and photovoltaic systems are being released at large scales to generate power (Noxpanco et al. 2020). So, modeling and simulation of PV systems are important ways to provide accurate predictions about cell temperature and energy production under various environmental conditions. The main problem for the solar cell is that high temperature or current density can damage the cell and reduce PV output power and electrical efficiency. So, many studies were developed in the last decades to enhance both the electrical and thermal performance of the PV panel by decreasing the operating temperature of the solar cells by using various techniques of the cooling system. For instance, Soliman and Hassan (2018a, b) proposed a 3D model for a PV system coupled with a microchannel, and the thermal model was solved based on the finite volume method using Ansys software. The findings show that using a microchannel in the PV system leads to a decrease in the cell temperature of PV by nearly 15 °C. Reddy et al. (2014) investigated how a micro-channel heat sink cooling system affects solar cell performance and identified the optimal configuration to be 0.5 mm in width with an eight aspect ratio.

However, most previous models presented to simulate the PV systems are solved using traditional methods in solving the differential equations (DEs). These kinds of classical models do not give an accurate prediction about the performance of the photovoltaic panels, and the percentage of error between the experimental and numerical results is usually high Zahra and Nasr (2019). So, the fractional differential equation (FDE) is a kind of non-local equation that proves its efficiency in modeling many numbers of applied systems in engineering and science. These kinds of fractional (DEs) succeed in describing hereditary and memorial properties of a variety of processes and materials, unlike the conventional integer-order DEs, which fail to capture relevant phenomena and do not accurately predict the PV performance. The field of fractional calculus plays a crucial role in many numbers of applications in signal and image processing, probability theory, biology, chemistry, communication, and control engineering, especially in heat transfer modeling (Abro et al. 2019; Yang et al. 2022; Abdalla et al. 2022; Sweilam et al. 2021; Shah and Irfan 2020; Shukla and Sapra 2019; Saqib et al. 2019; Sun et al. 2019; Evans et al. 2017). For instance, Abro et al. (2019) suggested a fractional model to improve the process of heat transfer for some devices related to solar energy and show the influence of adding multi-wall carbon nanotubes (MWCNTs) on the rate of heat transfer. In modeling the process of metal laser drilling, Zahra et al. (2020) presented a new fractional model using a meshless method; the order of the derivative is taken as constant and variable. It is found that the use of variable order has the best matching with the experimental data in the two stages of both steady-state and transient than using fixed order. For modeling the 2-D heat transfer process in a thin rectangular metallic surface heated up by an electric heater, Oprzędkiewicz, Mitkowski, and Rosół (2021) proposed a new, state space, fractional-order model based on Caputo fractional operator to express the fractional-order differences along time through the rectangular surface. The theoretical results are validated using experimental data measured by a thermal camera, and it was found that the fractional model is more accurate than the integer-order one. Moreover, Žecová and Terpák (2015) built a 1-D heat conduction model by using integer and fractional derivatives for estimating the thermal diffusivity. The results proved that the accurate value of the thermal diffusivity mainly depends on the time step and the number of previous values of temperatures in time.

The majority of heat conduction models based on the Fourier law are suitable for numerous engineering applications. However, it fails to accurately predict system performance, particularly at short spatial and temporal scales, and the initial disturbance in the heat process is not sensible by the Fourier law; this phenomenon is known as the heat conduction paradox (HCP) (Wang and Li 2021). Therefore, many non-Fourier models have been proposed to compensate for this defect in temperature prediction and for getting a better description of the physical phenomena and applied systems. In recent decades, the Cattaneo model was proposed to overcome the problems resulting from Fourier law by taking into account the lagging time $\tau$ between the temperature gradient and the vector of heat flux. The Cattaneo heat transfer model attracts many scientists and engineers. Qi et al. (2013) presented a numerical study for the laser short-pulse heating of a solid surface using the fractional Cattaneo model to show the effect of relaxation time $\tau$ on the speed of heat conduction propagation. The results found that the speed of heat propagation is increasing by decreasing the value of $\tau$. Another numerical study using fractional Cattaneo subdiffusion model was introduced by Milad Mozafarifard et al. (2021) to show the rapid-transient process of heat stream in a porous material. The numerical outcomes exactly matched with the experimental data, which proves the capability of the Cattaneo model in analyzing the transient process in the porous medium at a small-time scale. The numerical approach for simulating the fractional heat conduction model in a porous medium using the Cattaneo model is also presented in Nikan et al. (2021). The fractional model is solved in the sense of Caputo at order 1 < *α* < 2, and a localized meshless algorithm to discretize the spatial derivative.

The investigation of the solar cell systems has not stopped on increasing the produced amount of PV output power and enhancing its efficiency, but it is also expanded to the works of exergy assessment which mainly depends on the second law of thermodynamics. Exergy evaluation is an important and a powerful examination tool for the optimization, design, and evaluation of different energy systems compared to energy analysis that applied the first law of thermodynamics. Therefore, due to the exclusive concept and importance of exergy analysis in determining the locations, and kinds of irreversibility and losses approaches of energy systems, the attention has been raised to estimate and augment the PV systems based on the exergy analysis (Abo-Elfadl et al. 2021; Yousef et al. 2017; Abd Elbar, Yousef, and Hassan 2019; Yousef and Hassan 2019). For instance, Miansari et al. (2020) introduced a numerical model for improving the performance of shell and tube heat exchangers based on energy and exergy analysis. Moreover, the steam cycle of Montazeri steam power plant is analyzed for each equipment by Ahmadi and Toghraie (2016) using energy and exergy assessment. The results found that, according to exergy studies, the boiler is the major exergy waster, losing 85.66% of the total exergy entering the cycle Abdalla et al. (2022), Elbar et al. (2019), Mozafarifard et al. (2020), Qi et al. (2013), Yousef et al. (2016, 2022).

The works on the PV systems have been developed and extended to the evaluation of the financial and environmental aspects of solar cell systems. The methodologies of environmental and economic analysis give a strong insight into the effectiveness cost and the ecological influence by recognizing the required tools in their working conditions (G. Ahmadi, Toghraie, and Akbari 2019; Bing Mei, Pouya Barnoon, Davood Toghraie b, Chia-Hung Su, Hoang Chinh Nguyen 2022). According to the above discussion, increasing attention has been given to employing exergoeconomic and exergoenvironmental methodologies for assessing solar distillation systems (Hassan et al. 2021).

In spite of the large number of studies presented for predicting and improving the performance of the solar cell, still searching for an accurate model which provides a perfect indication of the PV performance and expects its amount of output power. According to the literature review and to the author’s best knowledge, the majority of the PV models were derived based on the classical Fourier law that leads to a system of classical differential equations. These kinds of differential equations produce a significant error between the theoretical results and the actual experimental data. As a result, the main objective of the paper is divided into two major points for predicting and enhancing the PV performance: the first objective is to derive and solve a new fractional Cattaneo PV model in the sense of the Riemann–Liouville fractional operator. Then, a comparative study between the actual experimental results (Yousef et al. 2016), the results using the proposed fractional Cattaneo model, and the results in A. M. Soliman and Hassan (2018a, b and Gad et al. (2022) are introduced. Second, based on the proposed fractional model, the current study presents a new PV cooling technique for improving PV performance by combining two configurations of an aluminum heat spreader (rectangular and trapezoidal) with the PV panel. All of the proposed PV systems are compared in terms of their output power, electrical efficiency, exergy efficiency, cost, and environmental and social costs under different climate circumstances (Summer and Winter).

## Derivation of fractional Cattaneo PV model

The heat transfer model can be achieved by substituting the integer time derivative term with fractional order in the Fourier model as follows (Mozafarifard et al. 2020): 

1 $$\rho \mathrm{c}{\tau }^{\alpha -1}\frac{{\partial }^{\alpha }T(x,y,t)}{\partial {t}^{\alpha }}=k{\nabla }^{2}T(x,y,t)$$

where $\frac{{\partial }^{\alpha }}{\partial {t}^{\alpha }}(.)$ is the Riemann–Liouville fractional derivative.

Also, Eq. (1) can be presented as below:

2 $$\rho \mathrm{c}{\tau }^{\alpha -1}\frac{{\partial }^{\alpha }T(x,y,t)}{\partial {t}^{\alpha }}=-\nabla \cdot q(x,,y,t)$$

Cattaneo suggested the heat conduction equation by considering the lagging time $\tau$ between the temperature gradient and the vector of heat flux in the following form (Nikan et al. 2021; Mozafarifard, Toghraie, and Sobhani 2021):

3 $$q(x,y,t+\tau )=-k\nabla T(x,y,t)$$

Now, by using the formulation of the Taylor series to the first order,

4 $$q\left(x,y,t+\tau \right)=\left(1+\tau \frac{\partial }{\partial t}\right)q(x,y,t)$$

and then by substituting with Eqs. (3) in (4), which results in

5 $$-k\nabla T(x,y,t) =\left(1+\tau \frac{\partial }{\partial t}\right)q(x,y,t)$$

By taking the divergence of both sides of Eq. (5), we get

6 $$-k{\nabla }^{2}T\left(x,y,t\right)=\left(1+\tau \frac{\partial }{\partial t}\right)\nabla \cdot q(x,,y,t)$$

and by combining Eqs. (2) with (6), then the new fractional Cattaneo heat conduction is given by

7 $$-k{\nabla }^{2}T\left(x,y,t\right)=\left(1+\tau \frac{\partial }{\partial t}\right)(- \rho \mathrm{c}{\tau }^{\alpha -1}\frac{{\partial }^{\alpha }T\left(x,y,t\right)}{\partial {t}^{\alpha }})$$

or

8 $$k{\nabla }^{2}T\left(x,y,t\right)= \rho \mathrm{c}{\tau }^{\alpha -1}\frac{{\partial }^{\alpha }T\left(x,y,t\right)}{\partial {t}^{\alpha }}+ \rho \mathrm{c}{\tau }^{\alpha }\frac{{\partial }^{1+\alpha }T\left(x,y,t\right)}{\partial {t}^{1+\alpha }}$$

One can have:

$$\frac{{\partial }^{2}T(x,y,t)}{\partial {x}^{2}}+\frac{{\partial }^{2}T(x,y,t)}{\partial {y}^{2}}=\frac{\rho c}{k}\left[{\tau }^{\alpha -1}\frac{{\partial }^{\alpha }T\left(x,y,t\right)}{\partial {t}^{\alpha }}+ {\tau }^{\alpha }\frac{{\partial }^{\beta }T\left(x,y,t\right)}{\partial {t}^{\beta }}\right],0<\alpha <\mathrm{1,1}<\beta <2$$

Then our new model can be written as

9 $$\frac{{\partial }^{2}T(x,y,t)}{\partial {x}^{2}}+\frac{{\partial }^{2}T(x,y,t)}{\partial {y}^{2}}=\frac{\rho c}{k}\left[{\tau }^{\alpha -1}{{}_{0}{}^{RL}\mathfrak{D}}_{t}^{\alpha }\mathrm{T}+ {\tau }^{\alpha }{{}_{0}{}^{RL}\mathfrak{D}}_{t}^{\beta }\mathrm{T }\right], 0<\alpha <1, 1<\beta <2$$

where ${{}_{0}{}^{RL}\mathfrak{D}}_{t}^{r}$ represents the Riemann–Liouville fractional operator which will be defined in the next section.

Based on Eq. (9), the new fractional Cattaneo model for describing each layer of the PV coupled with HS can be presented as

10 $$\mathrm{for}\;\mathrm{glass}\;\mathrm{cover}\;T_g:\frac{\partial^2T_g\left(x,y,t\right)}{\partial x^2}+\frac{\partial^2T_g\left(x,y,t\right)}{\partial y^2}=\frac{\rho_gc_g}{k_g}\left[\tau^{\alpha-1}{}_0{}^{RL}\mathfrak D_t^\alpha T_g+\tau^\alpha{}_0{}^{RL}\mathfrak D_t^\beta T_g\right]+Q_g$$

11 $$\mathrm{for}\;\mathrm{top}\;\mathrm{EVA},\;T_{ET}:\frac{\partial^2T_{ET}\left(x,y,t\right)}{\partial x^2}+\frac{\partial^2T_{ET}\left(x,y,t\right)}{\partial y^2}=\frac{\rho_Ec_E}{k_E}\left[\tau^{\alpha-1}{}_0{}^{RL}\mathfrak D_t^\alpha T_{ET}+\tau^\alpha{}_0{}^{RL}\mathfrak D_t^\beta T_{ET}\right]+Q_{ET}$$

12 $$\mathrm{for}\;\mathrm{cell}\;\mathrm{layer}\;T_c:\frac{\partial^2T_c\left(x,y,t\right)}{\partial x^2}+\frac{\partial^2T_c\left(x,y,t\right)}{\partial y^2}=\frac{\rho_cc_c}{k_c}\left[\tau^{\alpha-1}{}_0{}^{RL}\mathfrak D_t^\alpha T_c+\tau^\alpha{}_0{}^{RL}\mathfrak D_t^\beta T_c\right]+Q_c$$

13 $${\mathrm{for}\;\mathrm{bottom}\;\mathrm{EVA}\;T}_{EB}:\frac{\partial^2T_{EB}\left(x,y,t\right)}{\partial x^2}+\frac{\partial^2T_{EB}\left(x,y,t\right)}{\partial y^2}=\frac{\rho_Ec_E}{k_E}\left[\tau^{\alpha-1}{}_0{}^{RL}\mathfrak D_t^\alpha T_{EB}+\tau^\alpha{}_0{}^{RL}\mathfrak D_t^\beta T_{EB}\right]+Q_{EB}$$

14 $${\mathrm{for}\;\mathrm{TPT}\;\mathrm{layer}\;T}_T:\frac{\partial^2T_T\left(x,y,t\right)}{\partial x^2}+\frac{\partial^2T_T\left(x,y,t\right)}{\partial y^2}=\frac{\rho_Tc_T}{k_T}\left[\tau^{\alpha-1}{}_0{}^{RL}\mathfrak D_t^\alpha T_T+\tau^\alpha{}_0{}^{RL}\mathfrak D_t^\beta T_T\right]+Q_T$$

15 $$\mathrm{for}\;\mathrm{HS}\;\mathrm{layer}\;T_{HS}:\frac{\partial^2T_{HS}\left(x,y,t\right)}{\partial x^2}+\frac{\partial^2T_{HS}\left(x,y,t\right)}{\partial y^2}=\frac{\rho_{HS}c_{HS}}{k_{HS}}\left[\tau^{\alpha-1}{}_0{}^{RL}\mathfrak D_t^\alpha T_{HS}+\tau^\alpha{}_0{}^{RL}\mathfrak D_t^\beta T_{HS}\right]+Q_{HS}$$

where $Q$ is the internal heat generation in each layer which is considered to be 0 in the EVA, TPT, and HS layers as they do not absorb any significant incident or reflected radiation. While the heat generation inside the glass cover ${Q}_{g}$ is described by the following equation (Aly et al. 2018a, b):

16 $${Q}_{g}=\frac{{\lambda }_{g}\times G\times {A}_{pv}}{{V}_{g}}$$

where ${\lambda }_{g}$ is the absorptivity of the glass that is assumed to be equal to 0.05, $G$ is the solar irradiance in W/m^2^, and ${A}_{pv}$ is the area of the photovoltaic panel in m.^2^. The internal heat generation in the cell layer PV cell ${Q}_{c}$ is calculated as (Aly et al. 2018a, b)

17 $${Q}_{c}={A}_{pv}(1-{\eta }_{c})\times G\times { \omega }_{c}\times {\lambda }_{g}$$

where ${\omega }_{c}$ is the transmissivity of the silicon.

**Fig. 1:**
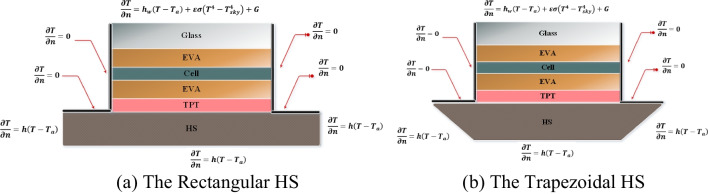
The schematic diagram for a photovoltaic system coupled with HS

The thermophysical properties of each layer of the PV panel are supposed to be constant and are presented in Table 1, while the material properties of the PV module (absorptivity, emissivity, transmissivity, and reflectivity) are illustrated in Table 2. This study aims to predict the temperature of the cell layer, which has a significant impact on the PV output (power, electrical efficiency, exergy efficiency). The study has been carried out by adding a heat spreader (HS) with a rectangular and trapezoidal shape which was not presented before in the PV solar system, as shown in Fig. 1.

**Table 1:** The thermophysical properties of the PV system

Layers	Width × Length (mm)	Thickness (mm)	Thermal conductivity (W/m.k)	Heat capacity (J/kg.K)	Density (kg/m^3^)
Glass	165 × 165	3	1.8	500	3000
EVA	165 × 165	0.15	0.35	2090	960
Silicon	165 × 165	0.225	130	677	2330
TPT	165 × 165	0.1	0.2	1250	1200
HS (Rec.)	600 × 600	10	273	900	2700
HS (Tra.)	600 × 165	10	273	900	2700

**Table 2:** The materials properties of the PV layers

Material layers	Absorptivity	Reflectivity	Transmissivity	Emissivity
Glass	0.04	0.04	0.92	0.85
EVA	0.08	0.02	0.9	-
Silicon	0.9	0.08	0.02	-
Tedlar	0.128	0.86	0.012	0.9

### Boundary and initial conditions 

The boundary conditions (BC) on the PV panel are considered as shown in Fig. 1. Firstly, the front surface is subjected to radiation and convection (Robin BC), so the boundary condition on the above surface is

18 $$\frac{\partial T}{\partial n}={h}_{w}\left(T-{T}_{a}\right)+\varepsilon \sigma \left({T}^{4}-{T}_{sky}^{4}\right)+G$$

where $\varepsilon$ is the emissivity of the surface, $\sigma$ is the Stefan Boltzman constant, $n$ is the normal direction to the surface, ${T}_{a}$ is the ambient temperature, and ${T}_{sky}$ is the temperature of the sky that is calculated by

19 $${T}_{sky}={T}_{a}-6$$

The convective heat transfer coefficient ${h}_{w}$ on the surface of the glass is obtained by

20 $${h}_{w}=5.82+4.07{v}_{w}$$

where ${v}_{w}$ is the wind velocity in m/s.

Secondly, the two sides of the PV panel are adiabatic surfaces that cannot absorb or lose heat to the environment (insulated surface) with Neumann BC:

21 $$\frac{\partial T}{\partial n}=0$$

Finally, the bottom surface and the two sides of the heat spreader are subjected to convection with Robin boundary conditions as follows:

22 $$\frac{\partial T}{\partial n}=h\left(T-{T}_{a}\right)$$

In our fractional model, the value of heat transfer coefficient $h$ at the top surface is assumed to be double the value of the coefficient ${h}_{w}$ at the bottom surface (Natural convection) (A. M. A. Soliman and Hassan (2018a, b).

The PV system with HS has the following initial conditions:

$$T\left(0,x,y\right)={T}_{0},\mathrm{and }\begin{array}{c}\\ \hspace{0.25em}\frac{\partial T\left(0,x,y\right)}{\partial t}=0\end{array}$$

where $${T}_{0}=$$ 298 K.

### The output of the PV panel

To examine the performance of the PV system, the electrical efficiency ${\eta }_{c}$ of the solar cell should be estimated as follows (Hedayatizadeh et al. 2013):

23 $${\eta }_{c}={\eta }_{ref}[1-{\gamma }_{ref} \left({T}_{c}-{T}_{ref}\right)]$$

The electrical output power of the panel is determined according to the following equation:

24 $${P}_{pv}={\eta }_{c}{\upomega }_{c} {A}_{pv} {\lambda }_{g} G$$

The reference efficiency ${\eta }_{ref}$ is considered to be equal to 12.5%. The exergy efficiency ${\eta }_{ex}$ is defined as follows (Yousef et al. 2019; Huang et al. 2021):

25 $${\eta }_{ex}=\frac{{E}_{{X}_{out}}}{{E}_{{X}_{in}}}=\frac{{E}_{{X}_{out}}}{{E}_{{X}_{sun}}}$$

According to Patela’s equation, the input exergy is described as the amount of power obtained by the PV module as can be presented as follows (Huang et al. 2021):

26 $${E}_{{X}_{in}}={A}_{PV }G\left[1-\frac{4}{3}\left(\frac{{T}_{a}}{{T}_{sun}}\right)+\frac{1}{3}{\left(\frac{{T}_{a}}{{T}_{sun}}\right)}^{4}\right]$$

The exergy output from the PV module can be described mathematically as

27 $${E}_{{X}_{out}}={E}_{{X}_{ele}}-{E}_{{X}_{th}}$$

where ${E}_{{X}_{ele}}$ is the electrical exergy, which is defined as the amount of electricity produced by the PV module in watts and presented by the following relation (Huang et al. 2021):

28 $${E}_{{X}_{ele}}={V}_{oc}\;{I}_{sc}\;FF$$

where ${V}_{oc}$ is the open-circuit voltage in volts and ${I}_{sc}$ is the short circuit current in amperes. The values of ${V}_{oc}$ and ${I}_{sc}$ that were used in the present model are found in Yousef et al. (2016). FF is the fill factor which is defined as the ratio between the maximum power, to the product of both short-circuit current and open-circuit voltage (Greulich et al. 2010):

29 $$FF=\frac{{V}_{mmp}\;{I}_{mpp}}{{V}_{oc} {I}_{sc}}$$

The thermal exergy is the amount of energy lost to the surrounding areas from the PV system in the shape of heat and can be calculated by (Kumar et al. 2020; Hakim, Handoyo, and Wullandari 2018)

30 $${E}_{{X}_{th}}=\left({h}_{conv}+{h}_{rad}\right) {A}_{PV } \left({T}_{a}-{T}_{c}\right) [1-\left(\frac{{T}_{a}}{{T}_{c}}\right)]$$

where ${h}_{conv}$ and ${h}_{rad}$ are the convection and radiation heat transfer coefficients.

### Economical analysis

To perform the economic analysis of the proposed photovoltaic systems, several parameters should be considered such as the capital cost of the PV (P_s_), annual maintenance cost (AMC), sinking fund factor (SFF), and annual salvage value (ASV). So, the cost of electricity production for the photovoltaic system can be assessed by (Gad et al. 2022; Yousef et al. 2022)

31 $${C}_{e}=\frac{UAC}{{E}_{{n}_{out}}}$$

where ${E}_{{n}_{out}}$ is the annual output power in kilowatt hours per year and UAC is the total operational annual cost of the PV system.

### Environmental evaluation

To show the effect of the modified PV systems on the environment as well as measure its environmental superiority against the other conventional energy sources, an environmental assessment should be performed based on computing the amount of the released CO_2_. As a result, the amount of CO_2_ mitigated per year is set as (Yousef et al. 2022)

32 $${\varnothing }_{{co}_{2}}=\frac{2({E}_{{n}_{out}}\times n)}{1000}$$

where ${\varnothing }_{{co}_{2}}$ is the environmental parameter. An enviroeconomic analysis is estimated according to the price of CO_2_ emitted over the lifetime of the PV and can be estimated as (Yousef et al. 2022)

33 $${Z}_{{co}_{2}}={{\varnothing }_{{co}_{2}}\times z}_{{co}_{2}}$$

$${Z}_{{co}_{2}}$$ is the enviro-economic parameter and $${z}_{{co}_{2}}$$ is the international carbon price, which is assumed to be 14.5 $ per ton of CO_2_.

## Numerical simulation

The proposed model is solved using two approximations of fractional derivative and nonstandard finite difference techniques in the temporal and spatial discretization, respectively.

### Temporal discretization

#### Definition 3.1

The Riemann Liouville fractional derivative (RL) for the function $T\left(t\right)$ is presented as (Sales Teodoro et al. 2019).

34 $$\begin{array}{cc}{{}_{0}{}^{RL}\mathfrak{D}}_{t}^{\alpha }T\left(t\right)=\frac{1}{\Gamma \left(n-\alpha \right)}\frac{{d}^{n}}{d{t}^{n}}{\int }_{0}^{t}{\left(t-u\right)}^{n-\alpha -1}T\left(u\right) du,& n-1< \end{array}\mathrm{\alpha } \le n$$

#### Definition 3.2

The shifted Grünwald-Letnikov (SGL) can be defined as (Sales Teodoro et al. 2019; Sun et al. 2019; Milici, Drăgănescu, and Tenreiro Machado 2019).

35 $${A}_{\Delta t,s}^{\alpha }T\left(t\right)={\left(\Delta t\right)}^{-\mathrm{\alpha }}{\sum }_{j=0}^{\infty }{g}_{j}^{\alpha } T\left(t-(j-s)\Delta t\right)$$

where $s$ is an integer number, and if the value of $s=0$, we back to the standard formula of GL; also, ${g}_{j}^{\alpha }$ represents the weight function which can be calculated by

$${{g}_{0}^{\alpha }=1, g}_{j}^{\alpha }=\left(1-\frac{\alpha +1}{j}\right){g}_{j-1}^{\alpha }, j\ge 1$$

Additionally, the coefficients ${g}_{j}^{\alpha }$ satisfy the following:

$${w}_{0}^{\alpha }=1, {w}_{1}^{\alpha }=-\alpha , {w}_{2}^{\alpha }\le {w}_{3}^{\alpha }\le {w}_{4}^{\alpha }\le \dots \le 0, \sum_{j=0}^{\infty }{w}_{j}^{\alpha }=0, \sum_{j=0}^{n}{w}_{j}^{\alpha }\ge 0, n\ge 1$$

Therefore, the first-order approximation of the RL fractional derivative can be rewritten as:

36 $$\begin{array}{cc}{{}_{0}{}^{RL}\mathfrak{D}}_{t}^{\alpha }T\left(t\right)={A}_{\Delta t,s}^{\alpha }T\left(t\right)+O(\Delta t),& n-1<\mathrm{\alpha } \le n\end{array}$$

two weighted shifted GL fractional operator (2-WSGD) as follows (Tian, Zhou, and Deng 2015; Zahra and Nasr 2019; Zahra et al. 2019):

37 $${{}_{0}{}^{RL}\mathfrak{D}}_{t}^{\alpha }T\left(t\right)=\frac{\alpha -2v}{2\left(\mu -v\right)}{A}_{\Delta t,\mu }^{\alpha }T\left(t\right)+\frac{2\mu -\alpha }{2(\mu -v)}{A}_{\Delta t,v}^{\alpha }T(t)+{O(\Delta t)}^{2}$$

where $\mu , v$, are integer numbers and $\mu \ne v$, and by choosing $\left(\mu , v\right)=(0, -1)$, then the previous equation can be described as:

38 $${{}_{0}{}^{RL}\mathfrak{D}}_{t}^{\alpha }T\left(t\right)=\left(1+\frac{\alpha }{2}\right){A}_{\Delta t,0}^{\alpha }T\left(t\right)-\frac{\alpha }{2}{A}_{\Delta t,-1}^{\alpha }T(t)+O\left({\Delta t}^{2}\right)$$

which can be written as follows:

39 $${{}_{0}{}^{RL}\mathfrak{D}}_{t,\Delta t}^{\alpha }T\left(t\right)={(\Delta t)}^{-\alpha }\sum_{j=0}^{\infty } {w}_{j}^{\alpha }T(t-j\Delta t)+O{(\Delta t)}^{2}$$

where $${w}_{0}^{\alpha }=\left(1+\frac{\alpha }{2}\right){g}_{0}^{\alpha };{ w}_{j}^{\alpha }=\left(1+\frac{\alpha }{2}\right){g}_{j}^{\alpha }-\frac{\alpha }{2}{g}_{j-1}^{\alpha }, j\ge 1$$.

### Spatial discretization

The nonstandard central difference approximation for the spatial derivatives can be discretized as:

40 $$\frac{{\partial }^{2}T}{\partial {x}^{2}}{|}_{{x}_{m}{,y}_{n},{t}_{p}}=\frac{{T}_{m+1,n}^{p}-2{T}_{m,n}^{p}+{T}_{m-1,n}^{p}}{{(\varnothing \left(\Delta x\right))}^{2}}+O({\varnothing \left(\Delta x\right)}^{2})$$

41 $$\frac{{\partial }^{2}T}{\partial {y}^{2}}{|}_{{x}_{m}{,y}_{n},{t}_{p}}=\frac{{T}_{m,n+1}^{p}-2{T}_{m,n}^{p}+{T}_{m,n-1}^{p}}{{(\delta (\Delta y))}^{2}}+O({\delta (\Delta y)}^{2})$$

by setting $\varnothing \left(\Delta x\right)=\Delta x$ and $\delta \left(\Delta y\right)=\Delta y$, we get the classical finite difference approximations (W. K. Zahra and Hikal 2017).

In the present model, the two functions of $\varnothing (\Delta x)$ and $\delta (\Delta y)$ are taken as

$$\begin{array}{cc}\Phi (\Delta x)=1-{e}^{-\Delta x}& \delta \left(\Delta y\right)=\mathrm{sin}\left(\Delta y\right)\end{array}$$

### Discretizing the PV system

The PV system given by Eqs. (10)–(15) can be discretized for any interior node using the two weighted shifted GL fractional approximation (2-WSGD) which presented in Eq. (39) at the point $\left({t}_{p}, {x}_{m},{y}_{n}\right)$ as.

for the glass layer:

42 $$\frac{{k}_{g}}{ {{\rho }_{g}{c}_{g}(\varnothing (\Delta x))}^{2}}\left({{T}_{g}}_{m+1,n}^{p-1}-{{T}_{g}}_{m,n}^{p-1}\right)+\frac{{k}_{g}}{ {{\rho }_{g}{c}_{g}(\varnothing (\Delta x))}^{2}}\left({{T}_{g}}_{m-1,n}^{p-1}-{{T}_{g}}_{m,n}^{p-1}\right)+\frac{{k}_{g}}{{{\rho }_{g}{c}_{g}(\delta \left(\Delta y\right))}^{2}}\left({{T}_{g}}_{m,n+1}^{p-1}-{{T}_{g}}_{m,n}^{p-1}\right)+\frac{{k}_{g}}{{{\rho }_{g}{c}_{g}(\delta \left(\Delta y\right))}^{2}}\left({{T}_{g}}_{m,n-1}^{p-1}-{{T}_{g}}_{m,n}^{p-1}\right)-\frac{{k}_{g}}{{\rho }_{g}{c}_{g}}{Q}_{g}={\tau }^{\alpha -1}(\Delta {t)}^{-\alpha }[{{T}_{g}}_{m,n}^{p}+{\sum }_{j=1}^{p}{w}_{j}^{\alpha } {{T}_{g}}_{m,n}^{p-j}] +{\tau }^{\alpha }(\Delta {t)}^{-\beta }[{{T}_{g}}_{m,n}^{p}+{\sum }_{j=1}^{p}{w}_{j}^{\beta } {{T}_{g}}_{m,n}^{p-j}]$$

for EVA layer:

43 $$\frac{{k}_{EVA}}{ {{\rho }_{EVA}{c}_{EVA}(\varnothing (\Delta x))}^{2}}\left({{T}_{EVA}}_{m+1,n}^{p-1}-{{T}_{EVA}}_{m,n}^{p-1}\right)+\frac{{k}_{EVA}}{ {{\rho }_{EVA}{c}_{EVA}(\varnothing (\Delta x))}^{2}}\left({{T}_{EVA}}_{m-1,n}^{p-1}-{{T}_{EVA}}_{m,n}^{p-1}\right)+\frac{{k}_{EVA}}{{{\rho }_{EVA}{c}_{EVA}(\delta \left(\Delta y\right))}^{2}}\left({{T}_{EVA}}_{m,n+1}^{p-1}-{{T}_{EVA}}_{m,n}^{p-1}\right)\frac{{k}_{EVA}}{{{\rho }_{EVA}{c}_{EVA}(\delta \left(\Delta y\right))}^{2}}\left({{T}_{EVA}}_{m,n-1}^{p-1}-{{T}_{EVA}}_{m,n}^{p-1}\right)-\frac{{k}_{EVA}}{{\rho }_{EVA}{c}_{EVA}}{Q}_{EVA}={\tau }^{\alpha -1}(\Delta {t)}^{-\alpha }[{{T}_{EVA}}_{m,n}^{p}+{\sum }_{j=1}^{p}{w}_{j}^{\alpha } {{T}_{EVA}}_{m,n}^{p-j}] +{\tau }^{\alpha }(\Delta {t)}^{-\beta }[{{T}_{EVA}}_{m,n}^{p}+{\sum }_{j=1}^{p}{w}_{j}^{\beta } {{T}_{EVA}}_{m,n}^{p-j}]$$

for cell layer:

44 $$\frac{{k}_{c}}{ {{\rho }_{c}{c}_{c}(\varnothing (\Delta x))}^{2}}\left({{T}_{c}}_{m+1,n}^{p-1}-{{T}_{c}}_{m,n}^{p-1}\right)+\frac{{k}_{c}}{ {{\rho }_{c}{c}_{c}(\varnothing (\Delta x))}^{2}}\left({{T}_{c}}_{m-1,n}^{p-1}-{{T}_{c}}_{m,n}^{p-1}\right)+\frac{{k}_{c}}{{{\rho }_{c}{c}_{c}(\delta \left(\Delta y\right))}^{2}}\left({{T}_{c}}_{m,n+1}^{p-1}-{{T}_{c}}_{m,n}^{p-1}\right)+\frac{{k}_{c}}{{{\rho }_{c}{c}_{c}(\delta \left(\Delta y\right))}^{2}}\left({{T}_{c}}_{m,n-1}^{p-1}-{{T}_{c}}_{m,n}^{p-1}\right)-\frac{{k}_{c}}{{\rho }_{c}{c}_{c}}{Q}_{c}={\tau }^{\alpha -1}(\Delta {t)}^{-\alpha }[{{T}_{c}}_{m,n}^{p}+{\sum }_{j=1}^{p}{w}_{j}^{\alpha } {{T}_{c}}_{m,n}^{p-j}] +{\tau }^{\alpha }(\Delta {t)}^{-\beta }[{{T}_{c}}_{m,n}^{p}+{\sum }_{j=1}^{p}{w}_{j}^{\beta } {{T}_{c}}_{m,n}^{p-j}]$$

for TPT layer:

45 $$\frac{{k}_{T}}{ {{\rho }_{T}{c}_{T}(\varnothing (\Delta x))}^{2}}\left({{T}_{T}}_{m+1,n}^{p-1}-{{T}_{T}}_{m,n}^{p-1}\right)+\frac{{k}_{T}}{ {{\rho }_{T}{c}_{T}(\varnothing (\Delta x))}^{2}}\left({{T}_{T}}_{m-1,n}^{p-1}-{{T}_{T}}_{m,n}^{p-1}\right)+\frac{{k}_{T}}{{{\rho }_{T}{c}_{T}(\delta \left(\Delta y\right))}^{2}}\left({{T}_{T}}_{m,n+1}^{p-1}-{{T}_{T}}_{m,n}^{p-1}\right)+\frac{{D}_{T}}{ {(\delta \left(\Delta y\right))}^{2}}\left({{T}_{T}}_{m,n-1}^{p-1}-{{T}_{T}}_{m,n}^{p-1}\right)-\frac{{k}_{T}}{{\rho }_{T}{c}_{T}}{Q}_{T}={\tau }^{\alpha -1}(\Delta {t)}^{-\alpha }[{{T}_{T}}_{m,n}^{p}+{\sum }_{j=1}^{p}{w}_{j}^{\alpha } {{T}_{T}}_{m,n}^{p-j}] +{\tau }^{\alpha }(\Delta {t)}^{-\beta }[{{T}_{T}}_{m,n}^{p}+{\sum }_{j=1}^{p}{w}_{j}^{\beta } {{T}_{T}}_{m,n}^{p-j}]$$

for HS layer:

46 $$\frac{{k}_{HS}}{{{\rho }_{HS}{c}_{HS}(\varnothing (\Delta x))}^{2}}\left({{T}_{HS}}_{m+1,n}^{p-1}-{{T}_{HS}}_{m,n}^{p-1}\right)+\frac{{k}_{HS}}{{{\rho }_{HS}{c}_{HS}(\varnothing (\Delta x))}^{2}}\left({{T}_{HS}}_{m-1,n}^{p-1}-{{T}_{HS}}_{m,n}^{p-1}\right)+\frac{{k}_{HS}}{{{\rho }_{HS}{c}_{HS}(\delta \left(\Delta y\right))}^{2}}\left({{T}_{HS}}_{m,n+1}^{p-1}-{{T}_{HS}}_{m,n}^{p-1}\right)+\frac{{k}_{HS}}{{{\rho }_{HS}{c}_{HS}(\delta \left(\Delta y\right))}^{2}}\left({{T}_{HS}}_{m,n-1}^{p-1}-{{T}_{HS}}_{m,n}^{p-1}\right)-\frac{{k}_{HS}}{{\rho }_{HS}{c}_{Hs}}{Q}_{HS}={\tau }^{\alpha -1}(\Delta {t)}^{-\alpha }[{{T}_{HS}}_{m,n}^{p}+{\sum }_{j=1}^{p}{w}_{j}^{\alpha } {{T}_{HS}}_{m,n}^{p-j}] +{\tau }^{\alpha }(\Delta {t)}^{-\beta }[{{T}_{HS}}_{m,n}^{p}+{\sum }_{j=1}^{p}{w}_{j}^{\beta } {{T}_{HS}}_{m,n}^{p-j}]$$

### Applying the boundary conditions (BC)

According to the energy balance method in transient case (Aly et al. 2018a, b) to estimate the value of any node on the boundaries,

47 $${\dot{E}}_{in}+{\dot{E}}_{g}={\dot{E}}_{st}$$

The value of any node on the glass surface (top surface) that is subjected to convection and radiation with Robin BC is calculated by

48 $$\frac{{k}_{g}}{{\rho }_{g}{c}_{g} {(\varnothing (\Delta x))}^{2}}\left({{T}_{g}}_{m+1,n}^{p-1}-{{T}_{g}}_{m,n}^{p-1}\right)+\frac{{k}_{g}}{{\rho }_{g}{c}_{g}{(\varnothing (\Delta x))}^{2}}\left({{T}_{g}}_{m-1,n}^{p-1}-{{T}_{g}}_{m,n}^{p-1}\right)+\frac{{k}_{g}}{{\rho }_{g}{c}_{g}{(\delta \left(\Delta y\right))}^{2}}\left({{T}_{g}}_{m,n-1}^{p-1}-{{T}_{g}}_{m,n}^{p-1}\right)+\frac{2{h}_{w}}{{\rho }_{g}{c}_{g}\delta (\Delta y)}\left({ T}_{a}-{{T}_{g}}_{m,n}^{p-1}\right)+\frac{2\varepsilon \sigma }{{\rho }_{g}{c}_{g}\delta \left(\Delta y\right)}\left({T}_{sky}^{4}-{{({T}_{g}}_{m,n}^{p-1})}^{4}\right)+\frac{2}{{\rho }_{g}{c}_{g}}G\varnothing \left(\Delta x\right)=\frac{{{k}_{g}Q}_{g}}{{\rho }_{g}{c}_{g}}+{\tau }^{\alpha -1}(\Delta {t)}^{-\alpha }[{{T}_{g}}_{m,n}^{p}+{\sum }_{j=1}^{p}{w}_{j}^{\alpha } {{T}_{g}}_{m,n}^{p-j}] +{\tau }^{\alpha }(\Delta {t)}^{-\beta }[{{T}_{g}}_{m,n}^{p}+{\sum }_{j=1}^{p}{w}_{j}^{\beta } {{T}_{g}}_{m,n}^{p-j}]$$

Based on the energy balance method, the nodes on the bottom surface which subjected to convection are estimated as follows:

49 $$\frac{{k}_{HS}}{{\rho }_{HS}{c}_{HS} {(\varnothing (\Delta x))}^{2}}\left({{T}_{HS}}_{m+1,n}^{p-1}-{{T}_{HS}}_{m,n}^{p-1}\right)+\frac{{k}_{HS}}{{\rho }_{HS}{c}_{HS} {(\varnothing (\Delta x))}^{2}}\left({{T}_{HS}}_{m-1,n}^{p-1}-{{T}_{HS}}_{m,n}^{p-1}\right)+\frac{{k}_{HS}}{{\rho }_{HS}{c}_{HS}{(\delta \left(\Delta y\right))}^{2}}\left({{T}_{HS}}_{m,n+1}^{p-1}-{{T}_{HS}}_{m,n}^{p-1}\right)+\frac{2h}{{\rho }_{HS}{c}_{HS}\delta (\Delta y)}\left({ T}_{a}-{{T}_{HS}}_{m,n}^{p-1}\right)=\frac{{{k}_{HS}Q}_{HS}}{{\rho }_{HS}{c}_{HS}}+{\tau }^{\alpha -1}(\Delta {t)}^{-\alpha }[{{T}_{HS}}_{m,n}^{p}+{\sum }_{j=1}^{p}{w}_{j}^{\alpha } {{T}_{HS}}_{m,n}^{p-j}] +{\tau }^{\alpha }(\Delta {t)}^{-\beta }[{{T}_{HS}}_{m,n}^{p}+{\sum }_{j=1}^{p}{w}_{j}^{\beta } {{T}_{HS}}_{m,n}^{p-j}]$$

The nodes on the right insulated surface are calculated by the following equation:

50 $$\frac{k}{\rho c {(\varnothing (\Delta x))}^{2}}\left({T}_{m+1,n}^{p-1}-{T}_{m,n}^{p-1}\right)+\frac{k}{\rho c {(\varnothing (\Delta x))}^{2}}\left({T}_{m,n+1}^{p-1}-{T}_{m,n}^{p-1}\right)+\frac{k}{\rho c {(\delta \left(\Delta y\right))}^{2}}\left({T}_{m,n-1}^{p-1}-{T}_{m,n}^{p-1}\right)=\frac{kQ}{\rho c}+{\tau }^{\alpha -1}(\Delta {t)}^{-\alpha }[{T}_{m,n}^{p}+{\sum }_{j=1}^{p}{w}_{j}^{\alpha } {T}_{m,n}^{p-j}] +{\tau }^{\alpha }(\Delta {t)}^{-\beta }\left[{T}_{m,n}^{p}+{\sum }_{j=1}^{p}{w}_{j}^{\beta } {T}_{m,n}^{p-j}\right]$$

The nodes on the left insulated surface are calculated by the following equation:

51 $$\frac{k}{\rho c {(\varnothing (\Delta x))}^{2}}\left({T}_{m-1,n}^{p-1}-{T}_{m,n}^{p-1}\right)+\frac{k}{\rho c {(\varnothing (\Delta x))}^{2}}\left({T}_{m,n+1}^{p-1}-{T}_{m,n}^{p-1}\right)+\frac{k}{\rho c {(\delta \left(\Delta y\right))}^{2}}\left({T}_{m,n-1}^{p-1}-{T}_{m,n}^{p-1}\right)=\frac{kQ}{\rho c}+{\tau }^{\alpha -1}(\Delta {t)}^{-\alpha }[{T}_{m,n}^{p}+{\sum }_{j=1}^{p}{w}_{j}^{\alpha } {T}_{m,n}^{p-j}] +{\tau }^{\alpha }(\Delta {t)}^{-\beta }\left[{T}_{m,n}^{p}+{\sum }_{j=1}^{p}{w}_{j}^{\beta } {T}_{m,n}^{p-j}\right]$$

Equations (44) and (45) can be used for any insulated node on the left and right surfaces of the PV panel, respectively, just by changing the thermophysical properties according to the material of each layer.

## Results and discussions

This section is divided into two parts. Firstly, PV model validation where the results of the non-Fourier (Cattaneo) fractional model, the results of the Fourier fractional model, and the results obtained by the classical models of A. M. Soliman and Hassan (2018a, b) and Gad et al. (2022) are compared with real experimental data under hot weather conditions (Yousef et al. 2016) in Egypt. Secondly, the effect of using HS on the PV system is discussed under various climatic conditions.

### Validation of the PV model

The validation of the proposed Fourier and non-Fourier models is accomplished by comparing the numerical solution with experimental and theoretical results obtained from the literature under the same conditions of ambient temperature and solar radiation.

#### Experimental validation

**Fig. 2:**
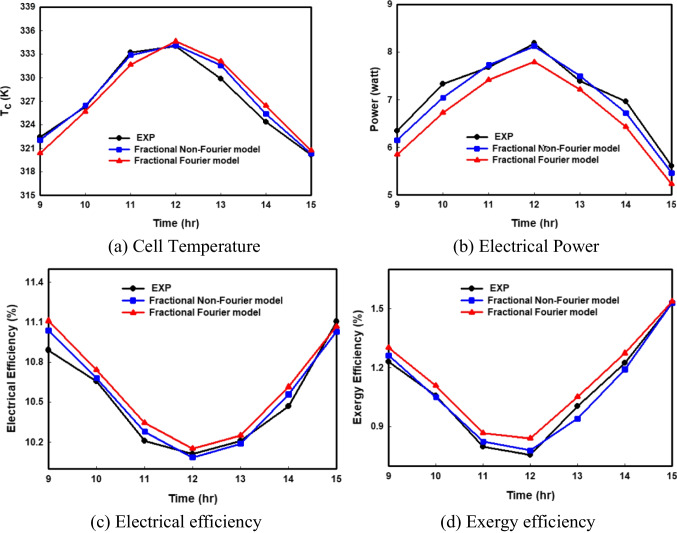
Comparison of the hourly variations in PV output using the non-Fourier model, Fourier model, and experimental data (Yousef et al. 2016)

The experimental validation is performed by comparing the results of our fractional Cattaneo model with Yousef’s experimental results (Yousef et al. 2016) which are introduced in Fig. 2. From Fig. 2, it is found that the outcomes of the fractional Cattaneo model are more consistent with the measured data than the results of the Fourier model in the whole-time domain. The temperature curves of the Fourier model are less matched with the experimental results with a maximum value in cell temperature of nearly 334.62 K against 334.12 K resulting from the fractional Cattaneo model, while the maximum value measured from the experiment is 334.04 K. The reason lies in the fact that the non-local and non-singular kernel features, along with memory influence, are considered by the fractional Cattaneo model. The validation is also presented for the PV power, electrical efficiency, and exergy efficiency in Fig. 2b, 2c, and 2d, respectively. It is found that the percentages of error results from the fractional Cattaneo model are 0.628%, 0.245%, and 3.84% for the PV power, electrical efficiency, and exergy efficiency, respectively, while these percentages reached 4.76%, 0.437%, and 5.77% using the fractional Fourier model. These results prove the perfect validity of the non-Fourier model for predicting PV performance.

#### Theoretical validation

**Fig. 3:**
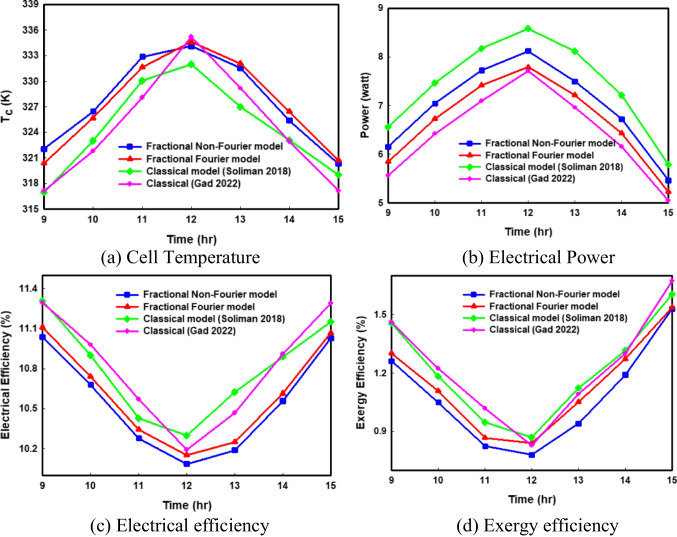
Comparison of the hourly variations in PV output using the non-Fourier model, Fourier model, and classical model (Gad et al. 2022; A. M. Soliman and Hassan (2018a, b)

The theoretical validation is performed by comparing the values of cell temperature, output power, electrical efficiency, and exergy efficiency estimated by the fractional Cattaneo model, and the Fourier model with their corresponding values solved by classical models (A. M. Soliman and Hassan (2018a, b; Gad et al. 2022) as can be shown in Fig. 3. The outcomes show that there is a significant difference between the experimental curve and the numerical curve solved by the classical models during the whole period of measurement. In the case of the greatest value of exergy efficiency, it is noticed that the error results from the classical models of Gad et al. (2022) and A. M. Soliman and Hassan (2018a, b) reached 13.13% and 18.34%, respectively, against only 3.84% of error resulted from the Cattaneo (non-Fourier model). The outcomes of both theoretical and experimental validations are summarized in Table 3.

**Table 3:** Comparison of the present models, with Yousef et al. (2016), A. M. (Soliman and Hassan (2018a, b), and Gad et al. (2022)

PV models	Fractional proposed models		Classical models	
	Non-Fourier model	Fourier model	Classical model (A. M. Soliman and Hassan (2018a, b)	Classical model (Gad et al. 2022)
Percentage error in Tc (%)	0.025	0.175	0.61	0.354
Percentage error in power (%)	0.628	4.76	4.88	5.72
Percentage error in electrical efficiency (%)	0.245	0.437	1.56	0.745
Percentage error in exergy efficiency (%)	3.84	5.77	18.34	13.13

### Impact of using the HS on the PV system

Reducing the cell temperature can be passively accomplished by coupling an aluminum heat spreader at the bottom of the PV panel. This kind of HS has higher thermal conductivity. Besides, this cooling system does not need any additional supplementary power. Based on the previous outcomes, the best fractional model for the photovoltaic panel provides less error and is more consistent with the experimental data is Cattaneo one. So, all the following results are estimated based on the proposed fractional Cattaneo model.

#### Cell temperature

**Fig. 4:**
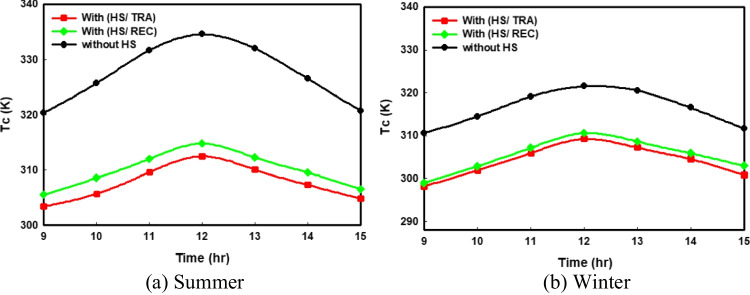
Comparison of the hourly changes in cell temperature for all the proposed PV systems

A comparison of the cell temperature in the case of utilizing rectangular HS and trapezoidal HS cooling techniques against the conventional PV system in the summer and winter seasons is presented in Fig. 4a and 4b, respectively. As presented in the two figures, if we compare the curves of cell temperatures, we can observe great differences between the values of cell temperature with and without using a heat spreader by maximum difference reaches nearly 20 K in summer and 12 K in winter. These significant differences in the cell temperature result from adding the aluminum spreader with high thermal conductivity, which helps in reducing the PV temperature module. According to Fig. 4a, it is noticed that the values of *T*_c_ in the case of trapezoidal HS are less than their corresponding values at using rectangular HS, with a maximum value (at 12 p.m.) of 314 K at the rectangular shape and 312 K at using the trapezoidal.

#### PV power

**Fig. 5:**
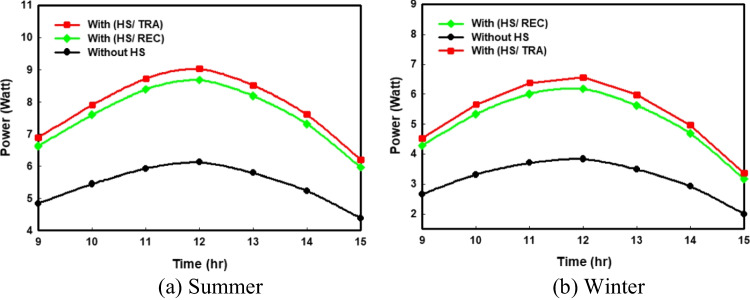
Comparison of the hourly variations of output power for all the proposed PV systems

The output power for the PV system is affected by the incident solar energy and the values of cell temperature. Figure 5a and 5b displays the hourly variations in output power with time in different cases (with and without HS) in summer and winter, respectively. Figure 5 shows that the produced output power in winter is less than in summer; this could be because the high solar intensity may have a significant impact on the performance of the PV system and hence increases its output from the electrical power. The reduction in the values of cell temperature is accomplished by increasing the PV output power. So, it is clear from Fig. 5a that adding HS to the PV panel increases its output power to reach 9.02 W with a trapezoidal shape and 8.68 W with a rectangular shape in the summer season, while the maximum value of the power without HS is 6.12 W.

#### Electrical efficiency

**Fig. 6:**
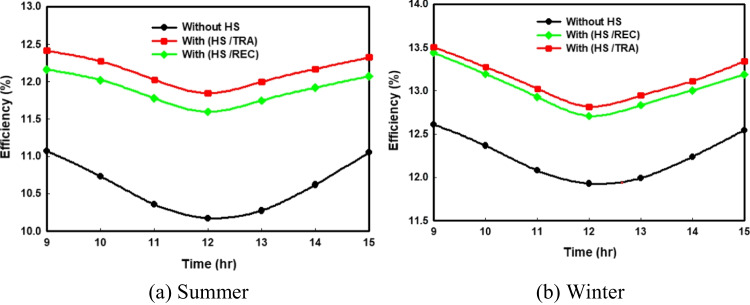
Comparison of the hourly variations of electrical efficiency for all the proposed PV systems

The hourly change of electrical efficiency with and without using the two shapes of HS in hot and cold climate circumstances is demonstrated in the Fig. 6a and 6b, respectively. The minimum value of the efficiency is accomplished at the solar noon due to the high losses at that time. Based on Fig. 6, it is found that the cooling system of the PV by HS improves its efficiency from about 10 to 12% in the summer season, while in winter, adding the trapezoidal and rectangular HS increases the PV efficiency to reach 7.53% and 6.7%, respectively.

#### Exergy efficiency

**Fig. 7:**
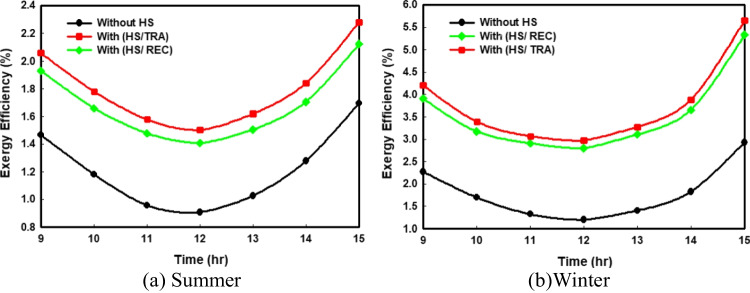
Comparison of the hourly variations of exergy efficiency for all the proposed PV systems

Exergy efficiency is an efficient tool to estimate the actual locations, types, and magnitudes of the losses and irreversibilities in the PV system. So, Fig. 7 illustrates the values of the exergy efficiency with and without using HS in the summer and winter times. As can be shown in Fig. 7, the values of exergy efficiency are less than their corresponding values of energy efficiency during the whole period of measurement; this is because, in the exergy analysis, all losses and irreversibilities are considered. Figure 7 displays a great enhancement in the exergy efficiency curves with and without using HS under various climate conditions. Furthermore, utilizing the HS with a rectangular shape is less effective than a trapezoidal spreader on the PV performance. The minimum value of exergy efficiency in summer due to natural convection is about 0.906% (without HS), and this percentage increased to nearly 1.5% and 1.4% after using trapezoidal and rectangular spreaders, respectively. In contrast, the previous percentage in the wintertime reached 1.2% without cooling and about 2.97% after passive cooling with trapezoidal HS.

### Cost analysis

In this analysis, the estimation of the cost electricity of power production has been recognized in three cases: the conventional PV panel, the PV system with rectangular HS, and the PV with the trapezoidal spreader. Each system is assessed at a rate of interest equal to 10% and at the lifetime of year 20 as can be presented in Table 4. As can be represented in Table 4, the outcomes revealed that the modified PV system with rectangular and trapezoidal HS accomplished a lower cost of electrical production compared to the traditional PV system. The cost of electrical production *C*_e_ for the PV system with rectangular and trapezoidal spreaders reached only 0.272 and 0.0.214 $/kWh, respectively, against 0.286 $/kWh for the PV without spreader. These results clearly indicate that the PV/HS is more economical than the conventional PV system. This is due to the higher electricity production after adding the spreader, compared to the initial cost of the system. Therefore, the best economic scenario could be obtained by integrating the PV panel with the trapezoidal spreader.

**Table 4:** Cost analysis for the PV system with and without HS

System	$${P}_{s}$$ ($)	CRF ($)	FAC ($)	AMC ($)	SSF ($)	ASV ($)	UAC ($)	$${E}_{{n}_{out}}$$ (KWh/year)	$${E}_{{X}_{out}}$$ (KWh/ year)	$${C}_{e}$$ $/ KWh	$${U}_{ex}$$ KWh /$
PV	30	0.117	3.52	0.528	0.0174	0.1044	3.94	13.76	16.2	0.286	4.11
PV/Rec.HS	40	0.117	4.68	0.702	0.0174	0.1392	5.24	19.26	22.8	0.272	4.35
PV/Tra.HS	32.7	0.117	3.83	0.574	0.0174	0.1139	4.29	20.03	24.3	0.214	5.68

### Environmental and enviroeconomic analysis

In this section, an environmental assessment based on computing the amount of the reduction in CO_2_ due to using the conventional PV, PV with rectangular HS, and PV with trapezoidal spreader is performed. The estimations of the rate of the avoided CO_2_ for the suggested PV systems are presented in Table 5. The results revealed that the expected amount of CO_2_ reduction based on the energy principle may reach 0.5504, 0.7704, and 0.8012 tons for PV, PV with rectangular HS, and PV with the trapezoidal spreader, respectively. This is because the increase of the electrical output energy for the PV with spreader is greater than the increase of embodied energy due to the integration of HS which leads to higher CO_2_ mitigation. The values of the relevant amount of CO_2_ avoided according to the exergy assessment are 0.6484, 0.9128, and 0.9932 tons, respectively. According to the previous results, the modified PV systems with the spreader are likely to be greener than the conventional one. The cause for this consequence is that the energy benefits from PV/HS during its lifecycle are larger than the traditional system.

**Table 5:** Environmental and enviro-economic parameters for the PV with and without HS

Parameters	PV	PV/Rec.HS	PV/Tra.HS
Lifetime (years)	20	20	20
$${E}_{{n}_{out}}$$(kWh) annual	13.76	19.26	20.03
$${E}_{{n}_{out}}$$(kWh) lifetime	275.2	385.2	400.6
Environmental parameter (rate ton Co_2_)	0.5504	0.7704	0.8012
Enviroeconomic parameter (rate $)	7.98	11.17	11.61
$${E}_{{X}_{out}}$$(kWh) annual	16.21	22.82	24.38
$${E}_{{X}_{out}}$$(kWh) lifetime	324.2	456.4	496.6
Exergoenvironmental parameter (rate ton Co_2_)	0.6484	0.9128	0.9932
Exergoenviroeconomic parameter (rate $)	9.37	13.23	14.4

## Conclusions

 To improve the thermal performance of the PV system coupled with HS, a new generalization of the fractional non-Fourier (Cattaneo) model in the sense of the RL fractional operator is presented. The fractional Cattaneo PV model with HS is solved using the second-order approximation of GL fractional derivative and nonstandard finite difference techniques in the temporal and spatial discretization, respectively. The results of the presented fractional Cattaneo model are compared with other numerical and experimental results obtained from the literature. The findings demonstrate that the fractional Cattaneo model perfectly matches the actual data, with error percentages in PV power and exergy efficiency of only 0.628% and 3.84%, respectively, compared to 5.72% and 13.13% resulted in Gad et al. (2022), 4.88% and 18.34% resulted in A. M. Soliman and Hassan (2018a, b). Then, an aluminum heat spreader (HS) with rectangular and trapezoidal are combined with a PV system to enhance its output. The outcomes show that a 20 K summer reduction and a 12 K winter reduction in cell temperature resulted from the addition of a trapezoidal HS. The daily average power was increased by about 28% in hot weather and by 37% in cold weather as a result of the reduction in PV temperature. Furthermore, a comparative analysis based on energetic, exergetic, economic, and enviroeconomic has been conducted for all the proposed PV systems with and without HS. It has been found that the cost of electrical production $${C}_{e}$$ for the PV system with rectangular and trapezoidal spreaders reached only 0.272 and 0.214 $/kWh, respectively, against 0.286 $/kWh for the PV without a spreader. Results demonstrated that PV, PV with rectangle HS, and PV with the trapezoidal spreader have the potential to reduce CO_2_ by 0.5504, 0.7704, and 0.8012 tons, respectively, based on the energy concept. According to the previous outcomes, authors suggest to extend their work using different cooling techniques to enhance the output of the solar cell based on various fractional operators.

## Data Availability

Not applicable.

## Nomenclatures

*A*: Area in m^2^
*C*: Specific heat in J/kg.K
*G*: Incident solar irradiance in W/m^2^
*h*: Heat transfer coefficient in W/m^2^.K
*k*: Thermal conductivity in W/m.K
Nu: Nussle number
*T*: Temperature in K
*v*: Wind velocity in m/s
*V*: Volume of the layer in m^3^
Re: Reynold number
Pr: Prandtl number

## Greek symbols

*η*: Efficiency
*τ*: Lagging time
*ρ*: Density in kg/m^3^
*σ*: Steven Boltzmann constant
*λ*: Absorptivity
*α*: Order of fractional derivative (0 < *α* < 1)
*ε*: Emissivity
*β*: Order of fractional derivative (1 < *β* < 2)

## Subscripts

a: Ambient
c: Cell
Ex: Exergy
EB: Bottom EVA
ET: Top EVA
f: Fluid
g: Glass
oc: Open circuit
ref: Reference
sc: Short circuit
st: Stored
th: Thermal
w: Wind

## Abbreviations

EVA: Ethyl vinyl acetate
FF: Fill Factor
GL: Grünwald-Letnikov
HCP: Heat conduction paradox
HS: Heat spreader
RL: Riemann- Liouville
TPT: Tedlar polyster tedlar
WSGL: Weighted-shifted Grünwald-Letnikov
